# Single-patient trial of dietary protein restriction in kidney disease: a patient-centred opportunity

**DOI:** 10.1093/ckj/sfae070

**Published:** 2024-03-30

**Authors:** Vincenzo Bellizzi, Denis Fouque

**Affiliations:** Division of Nephrology and Dialysis, Dept Medical Sciences AORN “Sant'Anna e San Sebastiano” Hospital, Caserta, Italy; Dept of Nephrology Dialysis Nutrition, hospital Lyon Sud, Hospices civils de Lyon, University Claude Bernard Lyon 1, Lyon, France

To the Editor,

Chronic kidney disease (CKD) is a major cause of disability and death, and any effective treatment is needed. Dietary protein restriction is efficient to improve CKD and delay dialysis, however its use is controversial. Whether or not to recommend low protein diets (LPDs) reflects the contrast between guidelines [1] which are based on the ideal-world, and real-life where the patient adherence is poor which reduces the effectiveness [2]. Doctors are conflicted between applying guidelines in any situation or deciding not to propose LPDs because of low adherence; neither is right or wrong, but there might be a difference between efficacy and effectiveness. The Food and Drug Administration calls for new approaches to study the usefulness of therapies, considering that treatments for chronic diseases are lifelong and show their effects in the long-term, more than the length of a trial. Among innovative strategies, the patient-centred approach is promoted [3]; this study proposes such a strategy as a third option for LPDs, giving patients a chance to choose for themselves.

## CASE REPORT

This is a single-patient (n-of-1) trial [4], exploring the effectiveness and safety of LPDs in the very long term (33 years). An 11-year-old boy was discovered with nephronophthisis and initial impaired renal function. He received general dietary advices and energy prescription above 35 kcal/kg/day. Since adult age up to 45 years, incremental dietary protein reductions were proposed to delay the start of dialysis.

At 20 years, the creatinine clearance was 54 mL/min/1.73 m^2^ (CKD-3a), and he was prescribed a standard low-normal protein intake (0.8 g/kg/day; low sodium). At 26 years, creatinine clearance was 45 mL/min/1.73 m^2^ (CKD-3b), a standard low protein/sodium diet (0.6 g/kg/day, 2.3 g Na/day) was prescribed. At 36 years, the glomerular filtration rate (GFR) was reduced to 24 mL/min/1.73 m^2^ (CKD-4), and a vegan sVLPD (0.3 g/kg/day) with aproteic food (Flavis^®^, Dr Schär, Italy), calcium salt of essential amino acids and ketoanalogues (0.125 g/kg/day; Ketosteril^®^, Fresenius Kabi, Germany), iron, folic acid and B vitamins was started; the prescribed dietary energy has been above 30 kcal/kg/day.

Clinical, nutritional and renal parameters were collected for each of the four dietary periods. Renal function was measured by creatinine clearance, and protein intake was calculated as follows: [(daily urinary urea nitrogen, UUN + daily non-UUN)*6.25].

## RESULTS

**Figure 1:**
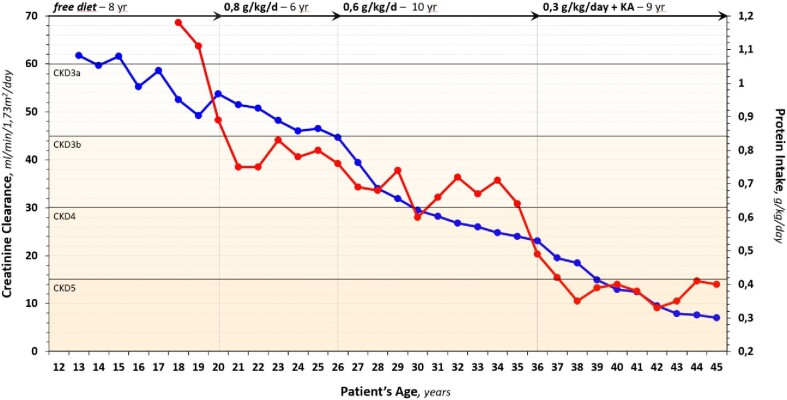
Protein intake (g/kg BW/day) in red and renal function (assessed by creatinine clearance, mL/min/1.73 m^2^) in blue plotted over time from the age of 12 until 45 years (light vertical lines and arrows at their top indicate the time-points of dietary protein prescription). GFR and protein intake data are the mean of at least two measurements during each year.

Initially (CKD-2/3a; 8 years in length), the boy was in growing phase, and the spontaneous protein intake was 1.2 g/kg/day; it was suggested to continue such diet and the protein intake during the period was 1.06 g/kg/day. During the next phases, on 0.8 (CKD-3a/b; 6 years), 0.6 (CKD-3b/4; 10 years) and 0.3 vegan (CKD-4/5; 9 years) protein diets, the mean protein intake was 0.78 (equal prescription), 0.68 and 0.49 (<0.2 above prescription), respectively. Adherence to each protein prescription along the follow-up was good; serum urea nitrogen, indeed, was around 25 mg/dL until CKD-3a and 35–40 in CKD-4/5 (Table 1 and Fig. 1).

**Table 1:** Clinical, nutritional and renal parameters along each different protein diets periods.

Protein prescription, g/kg/day	Free	0.8	0.6	0.3 + KA
Diet duration, years (pt age range, years)	8 (12–20)	6 (20–26)	10 (26–36)	9 (36–45)
Protein intake, g/kg/day	1.06 ± 0.15	0.78 ± 0.03	0.68 ± 0.04	0.49 ± 0.04
BUN, mg/dL	24 ± 4	25 ± 4	39 ± 12	36 ± 14
Systolic blood pressure, mmHg	124 ± 7	122 ± 3	128 ± 9	126 ± 9
Diastolic blood pressure, mmHg	79 ± 6	71 ± 6	74 ± 5	75 ± 5
Body weight, kg	58.7 ± 4.3	64.1 ± 1.9	67.5 ± 1.4	68.5 ± 1.0
BMI, kg/m^2^	20.6 ± 0.9	21.4 ± 0,7	22.5 ± 0.5	22.9 ± 0.3
Creatinine production, mg/kg/day	23 ± 2	22 ± 1	21 ± 2	18 ± 1
Lean body mass, kg	46 ± 4	48 ± 3	48 ± 4	44 ± 2
‘ , %BW	79 ± 6	74 ± 3	71 ± 6	64 ± 3
Creatinine clearance, mL/min/year	52.6 ± 8.5	47.9 ± 2.7	29.4 ± 5.0	17.5 ± 5.6
Δ Creatinine clearance, mL/min/year	−1.1	−1.5	−2.2	−1.9

All data are the mean ± standard deviation of all measurements along each dietary time period.

pt: patients; KA: ketoanalogues; BUN: blood urea nitrogen; BMI: body mass index.

Blood pressure was normal without medications along the whole time period. Enalapril was started at CKD-4 for proteinuria 1 g/day. Metabolic control during the follow-up was excellent. Serum levels of potassium, calcium, phosphorus, parathyroid hormone and haemoglobin were within normal range without medications. During the last 9-year period (CKD-4/5; GFR = 20 mL/min/1.73 m^2^) on 0.3 vegan diet, the mean serum values were: potassium 5.2 ± 0.4 mEq/L without binders; calcium 9.5 ± 0.6 mg/dL; phosphorus 4.2 ± 0.6 mg/dL without binders; and haemoglobin 11.9 ± 1.1 g/dL without ESA. Hyperuricemia and dyslipidemia were present at start; the patient was prescribed allopurinol and omega-3 fatty acids; a statin was regularly taken since CKD-3b.

Growth and nutritional status were well preserved. Height was 153 cm at 12 years and 173 cm at 18 years; weight rose from 48 to 66 kg at the same points. During the follow-up, body mass index (BMI) was stable at 22–23 kg/m^2^, and muscle mass was stable and adequate as proven by normal creatinine production and lean body mass. The patient exercised regularly (running, gym, soccer); during the CKD-4/5 on supplemented very low protein diet (sVLPD), the physical performance was 450–750 METs/week. The GFR decline rate was 1.7 mL/year during the whole period, with similar rates alongside each dietary regimen (Table 1 and Fig. 1).

Clinical, metabolic, nutritional and renal parameters were good until 1 month prior to starting dialysis (protein intake 0.40 g/kg/day; blood urea nitrogen 49 mg/dL; serum creatinine 8.66 mg/dL; serum potassium 5.2 mEq/L; serum calcium 9.5 mg/dL; serum phosphate 5.0 mg/dL; parathyroid hormone 409 pg/mL; haemoglobin 12.4 g/dL; weight 68.0 kg; BMI 22.7 kg/m^2^; creatinine production 18 mg/kg/day; lean body mass 43 kg—63% body weight (BW); creatinine clearance 7.0 mL/min; proteinuria 1.8 g/day); blood pressure was 170/110 mmHg, poorly responsive to drugs. The patient started incremental peritoneal dialysis.

## DISCUSSION

The apparent failure of the Modification of Diet in Renal Disease study to demonstrate the efficacy of an LPD to slow CKD progression was due to the initial GFR fall related to the acute haemodynamic effect of LPDs [5]. This initial GFR fall has hidden the long-term improvement due to the beneficial effect of low proteins on renal haemodynamics. An explanatory trial in very selected setting overcome this confounder by studying patients with proven adherence to LPD [6], demonstrating the efficacy of high adherence to LPDs to reduce GFR decline and delay dialysis start. Nowadays, innovative and less costly research approaches, focused on patients in whom the therapies will be used, such as pragmatic trials or long-lasting patient-centred outcome research, are strongly encouraged [3]. Furthermore, restricting the effects of LPDs to a GFR decline–centred view denies the well-known metabolic advantages of LPDs: lowering serum toxins, urea and phosphate, improving metabolic acidosis, anemia, CKD–mineral bone disease and blood pressure [7], allowing start of dialysis at lower GFR [8] (i.e. later) and better clinical conditions [9] (i.e. metabolic control).

A recent pragmatic trial in a real-life setting showed that only 33% of patients may adhere to an LPD (0.6 g/kg BW/day) and 23% to a sVLPD (0.3 g/kg BW/day) [10]. Because of low adherence, by intention-to-treat there was no difference between interventions; however, a per-protocol analysis suggested that in adherent patients the median time to renal death was 26 months in LPD and 54 months in sVLPD (*P *= .10), with hazard ratio for renal death of 0.65 (95% confidence interval 0.37–1.13) in sVLPD-adherent vs LPD-adherent patients. Hence, prescribing an sVLPD in unselected CKD patients may not have significant advantages, but adherent patients may benefit from metabolic improvement and postponing dialysis start in a safe environment.

This 33-year-long, single-patient, observational trial [4] indicates that with personalized LPDs and patient concordance, it is possible to maintain high, long-lasting adherence to several protein intakes, allowing slow kidney disease progression, and excellent metabolic control and nutritional status. The key point is the patient's choice and the resulting adherence to diet, which allows control of disease progression and delay of start of dialysis in response to an optimal metabolic control. Accordingly, the patient him/herself and the patient–doctor relationship fortify the way, and are of great importance. On an individual patient–doctor basis, there is no need for additional research. The doctor will be able to check the kidney function, metabolic and body parameters during the follow-up, and improve/correct any clinical or biological abnormality. Dietary counselling/corrections will help in doing this accordingly. Unmet needs will be fulfilled.

In conclusion, this cheap single-patient study in renal nutrition is more informative than complex trials, or at least brings sufficient knowledge to proceed. Here the evidence is already before our eyes: the general ‘one size fits all’ approach for low-protein diets in CKD does not work; meanwhile the precision-nutrition, patient-centred approach definitively lifts the veil on the major power of LPDs’ effectiveness and safety in CKD [11, 12]. The key point is patient choice (not doctor choice), and the resulting adherence to diet, avoiding missing an opportunity for many renal patients. Unselected CKD patients deserve dietary support and personalized nutritional treatment. Unfortunately, there are no data about the degree of conviction of unselected doctors regarding LPDs’ effectiveness. In renal nutrition, the patient-centred approach is a great opportunity to improve CKD for many patients worldwide and to impact on the burden of end-stage kidney disease.

## PATIENT CONSENT

The patient gave informed consent to publish this paper.
